# Kinetic, Thermodynamic, and Crystallographic Studies of 2-Triazolylthioacetamides as Verona Integron-Encoded Metallo-β-Lactamase 2 (VIM-2) Inhibitor

**DOI:** 10.3390/biom10010072

**Published:** 2020-01-01

**Authors:** Yang Xiang, Yue-Juan Zhang, Ying Ge, Yajun Zhou, Cheng Chen, Weixiao Yuan Wahlgren, Xiangshi Tan, Xi Chen, Ke-Wu Yang

**Affiliations:** 1 Key Laboratory of Synthetic and Natural Functional Molecule Chemistry of Ministry of Education, Chemical Biology Innovation Laboratory, College of Chemistry and Materials Science, Northwest University, Xi’an 710127, China; xiangyanglovelove@163.com (Y.X.);; 2 School of Physical Education, Yan’an University, Yan’an 716000, China; 3 Department of Chemistry and Institutes of Biomedical Sciences, Fudan University, Shanghai 200433, China; 4 Department of Chemistry and Molecular Biology, University of Gothenburg, Box 462, S-40530 Gothenburg, Sweden

**Keywords:** antibiotic resistance, metallo-β-lactamase VIM-2 inhibitor, 2-triazolylthioacetamides, thermodynamics, crystallographic study

## Abstract

Inhibition of β-lactamases presents a promising strategy to restore the β-lactams antibacterial activity to resistant bacteria. In this work, we found that aromatic carboxyl substituted 2-triazolylthioacetamides **1a**–**j** inhibited VIM-2, exhibiting an IC_50_ value in the range of 20.6–58.6 μM. The structure-activity relationship study revealed that replacing the aliphatic carboxylic acid with aromatic carboxyl improved the inhibitory activity of 2-triazolylthioacetamides against VIM-2. **1a**–**j** (16 mg/mL) restored the antibacterial activity of cefazolin against *E. coli* cell expressing VIM-2, resulting in a 4–8-fold reduction in MICs. The isothermal titration calorimetry (ITC) characterization suggested that the primary binding 2-triazolylthioacetamide (**1b**, **1c**, or **1h**) to VIM-2 was a combination of entropy and enthalpy contributions. Further, the crystal structure of VIM-2 in complex with **1b** was obtained by co-crystallization with a hanging-drop vapour-diffusion method. The crystal structure analysis revealed that **1b** bound to two Zn(II) ions of the enzyme active sites, formed H-bound with Asn233 and structure water molecule, and interacted with the hydrophobic pocket of enzyme activity center utilizing hydrophobic moieties; especially for the phenyl of aromatic carboxyl which formed π-π stacking with active residue His263. These studies confirmed that aromatic carboxyl substituted 2-triazolylthioacetamides are the potent VIM-2 inhibitors scaffold and provided help to further optimize 2-triazolylthioacetamides as VIM-2 even or broad-spectrum MβLs inhibitors.

## 1. Introduction

β-lactam antibiotics, including penicillins, cephalosporins, and carbapenems, are the clinically most important and commonly used antimicrobial agents [1]. However, the overuse of the β-lactam antibiotics has resulted in a rapid appearance of β-lactam-resistant bacteria [2]. A main mechanism for bacteria to develop resistance to β-lactams is the expression of β-lactamase, which hydrolyze the β-lactam ring and renders the antibiotics ineffective. According to the action mechanism, β-lactamases have been categorized as serine-β-lactamases (SβLs) and metallo-β-lactamases (MβLs) [3]. Based on amino acid sequence homology and Zn(II) content, MβLs are further divided into subclasses B1, B2, and B3. MβLs belonging to B1 and B3 subgroups hydrolyze almost all β-lactams [3]. The SβLs inhibitors, such as clavulanic acid, tazobactam, sulbactam, vaborbactam, and avibactam have been used clinically [4,5]. Although a large number of MβL inhibitors have been reported, including thiols [6,7,8,9], carboxylic acids [10,11], rhodanine [12,13,14], cyclic boronates [15,16], and ebselen [17,18], there are no MβL inhibitors for available clinical purposes to date. Consequently, it is an urgent need to develop novel MβL inhibitors and to investigate their action mechanism.

VIM-2, a B1 subclass MβL [19], was initially discovered in *P. aeruginosa*, which showed a high prelevance and broad substrate spectrum, including penicillins, cephalosporins, and carbapenems [20], it therefore reflects a significant drug target for the treatment of antibiotic-resistant bacterial infection. The crystal structure of VIM-2 has been reported [21,22]. Moreover, several complexes of crystal structures of VIM-2 with small molecules have been reported [7,11,13].

Recently, we found that azolylthioacetamides are the potent MβL inhibitors [23,24,25,26], these compounds showed broad-spectrum inhibition on CcrA [24], NDM-1 [23,24,26], ImiS [24,25,26], and L1 [24], as the representative of three subclasses enzymes, respectively. Further, we reported an aromatic carboxyl substituted azolylthioacetamide as an inhibitor of VIM-2, which provided the binding via the complex crystal structure of enzyme and inhibitor [27]. 

In this work, we synthesized a series of 2-triazolylthioacetamides as previously reported [25], tested their inhibitory activity against VIM-2, and evaluated their efficiency to restore antibacterial activity of cefazolin against bacteria expressing VIM-2. Further, the binding affinities of 2-triazolylthioacetamides to VIM-2 were determined by isothermal titration calorimetry (ITC). Finally, the crystal structure of VIM-2 in complex with a 2-triazolylthioacetamide molecule was determined and the interaction between enzyme and inhibitor was explored by crystallographic studies.

## 2. Materials and Methods 

### 2.1. Synthesis of 2-Triazolylthioacetamides

**Scheme 1:**
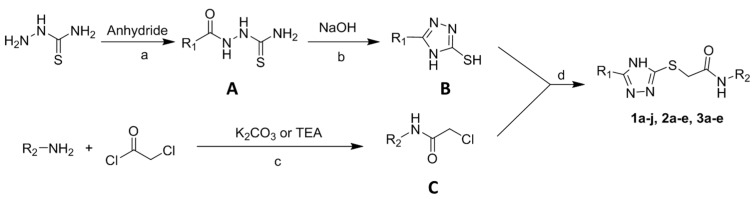
Synthetic route of the 2-trizolylthioacetamides.

The 2-triazolylthioacetamides were synthesized as shown in Scheme 1. Briefly, thiosemicarbazide reacted with corresponding acid anhydride (phthalic anhydride, succinic anhydride, and glutaric anhydride) to give thiosemicarbazides. The thiosemicarbazides was refluxed in NaOH aqueous to offer the thiol triazoles. Corresponding anilines were acylated with chloroacetyl chloride to offer α-chloroacetamides. Finally, the thiol triazoles cross-linked with α-chloroacetamide to afford the 2-triazolylthioacetamides under alkaline environment, (**1a**–**j**, **2a**–**e**, and **3a**–**e**).

### 2.2. Over-Expression and Purification of VIM-2

The VIM-2 was overexpressed and purified as previously described [21]. In brief, *E. coli* BL21 (DE3) cells were transelected with plasmid pET24a-VIM-2. A 10 mL overnight culture of these cells in lysogeny broth (LB) was used to inoculate of M9 media containing kanamycin (25 μg/mL). The cells were allowed to grow at 37 °C with shaking until the optical density at 600 nm (OD_600_) reached 0.6–0.8. VIM-2 expression was induced with 1 mM IPTG at 25 °C overnight. Cells were collected by centrifugation and lysed by sonication. The supernatant collected by centrifugation was dialyzed three times at 4 °C and loaded on a Q-Sepharose column. Bound protein was eluted with a 0–500 mM NaCl gradient in 30 mM Tris, pH 7.6. The further purification of crude VIM-2 was run though a G75 column (GE Healthcare, Boston, MA, USA) and eluted with 20 mM Tris, pH 7.6, containing 150 mM NaCl, and 5% β-mercaptoethanol. Enzyme purity was analyzed by SDS-PAGE, and determined the concentration by measuring the absorption at 280 nm (extinction co-efficient: 39,000 M^−1^cm^−1^) using the Nanodrop 2000 c spectrophotometer (Thermo Scientific, Waltham, MA, USA).

### 2.3. Inhibition Studies

The percent inhibition (inhibition%, enzyme activity without inhibitor minus residual activity with 100 μM inhibitor) and IC_50_ (the inhibitor concentrations causing 50% decrease of enzyme activity) values were measured using a previously established enzyme-inhibition assay on an Agilent UV8453 UV−Vis spectrophotometer using 60 μM nitrocefin (monitoring at 482 nm) as a substrate. The 2-triazolylthioacetamides **1a**–**j**, **2a**–**e**, and **3a**–**e** were dissolved with DMSO and diluted with ddH_2_O. The VIM-2 and nitrocefin were prepared with a Tris buffer (30 mM, pH 7.6). The final concentration of DMSO inhibition experiments was below 1%. The IC_50_ values were determined in triplicate against VIM-2, where the inhibitor concentrations were varied between 0 and 120 µM, and final concentrations VIM-2 was 0.6 nM. The tested VIM-2 and inhibitors were premixed at room temperature (30 mM Tris, pH 7.6), and then the substrate in the same buffer was added to the mixture, the initial rates of nitrocefin hydrolysis were recorded at each inhibitor concentration. The percentage inhibition was calculated by the equation inhibition% = 1 − (*V*_i_/*V*_0_) and IC_50_ values were calculated by plotting the average inhibition% against inhibitor concentration and fitting of the data.

### 2.4. Antibacterial Activity Assays

The minimum inhibitory concentration (MIC) of antibiotics, both alone and in the presence of enzyme inhibitors, was determined using the broth micro-dilution method [28]. *E. coli* DH10B containing plasmid pBCSK-VIM-2 grown in MH medium to OD_600_ = 0.45 were used as inocula, after an 84-fold dilution to 1 × 10^5^ CFU/mL in MH medium. Cefazolin was dissolved in MH medium to prepare 1024, 512, 256, 128, 64, 32, 16, and 8 for *E. coli* producing VIM-2 stock solutions, respectively. Compounds **1a**–**j**, **2a**–**e**, and **3a**–**e** were dissolved in DMSO and diluted with MH media to prepare 64 µg/mL stock solutions. The 50 μL prepared stock solutions with different cefazolin concentrations were diluted with a 50 μL inhibitor stock solution, then 100 μL inoculum was added sequentially. The final concentration of both the inhibitor and cefazolin were a quarter of the stock solution concentrations. 

### 2.5. Evaluation of Binding Affinity

Binding affinity measurements were performed by ITC on a Malvern MicroCal-ITC200 at 298 K. The syringe stirring rate was 750 rpm and kept constant. A stock of VIM-2 and inhibitor (**1b**, **1c**, and **1h**) were prepared in Tris buffer (30 mM, pH 7.6) with 1% DMSO. The concentration of VIM-2 was 100 μM. Before the injections, the solution of enzyme and inhibitor were degassed by centrifugation (32,583× *g*, 5 min), respectively. Then, the prepared VIM-2 solution was transferred into the sample cell (210 μL), and the inhibitor (1 mM) in the syringe was titrated into the sample cell by 18 injections (0.5 μL for the first injection and 1.5 μL for the other 17 injections) with 120 s intervals per injection. The heat of dilution for the inhibitor was determined in the control experiment and subtracted from the integrated data before curve fitting [29]. The data were fitted and analyzed with single binding sites using Origin 7.0 software, provided with the instrument.

### 2.6. Crystallization, X-ray Data Collection, and Structure Determination

The complex crystal of **1b** and VIM-2 was obtained with co-crystallization by the hanging-drop vapor-diffusion method. The 10 mM inhibitor in a 5% DMSO Tris buffer and 14.9 mg/mL VIM-2 solution were pre-incubated overnight with a 1:1 ratio. The reservoir consisting of 20% polyethylene glycol (PEG) 8000, 0.1 M MES pH 6.5, 0.1 M magnesium acetate, and 3% DMSO solution and crystals appeared after 2–10 d. Crystals were transferred to 24% PEG 8000, 0.1 M MES pH 6.5, 0.3 M magnesium acetate, 3% DMSO, and 25% glycerol and flash-cooled in liquid nitrogen. Data were collected at the Shanghai Synchrotron Radiation Facility (SSRF) and processed using HKL 3000. The phase was solved by molecular replacement Phenix [30] and a previously published VIM-2 structure (PDB: 1ko3) [22] as a search model. Structure refinements were actualized by iterative rounds of the model building using Wincoot [31] and maximum-likelihood restrained refinement with Phenix. The PyMOL molecular graphics system was used to produce illustrations and to visualize interactions [32]. The numbering of all residues is used as a class B β-lactamase numbering system [33].

## 3. Results and Discussion

### 3.1. Synthesis of 2-Triazolylthioacetamides

**Figure 1:**
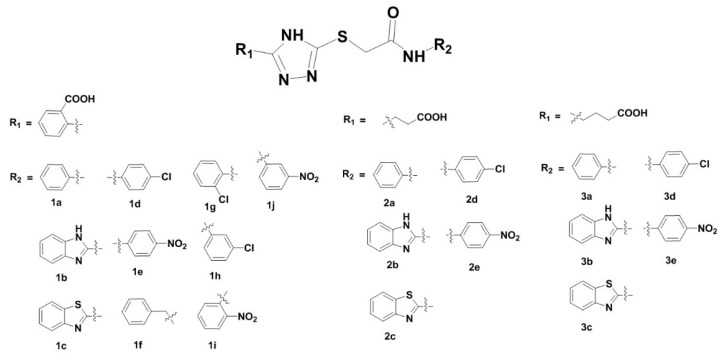
The structure of 2-triazolylthioactamides.

Compounds **1a**–**j**, **2a**-**e**, and **3a**-**e** were re-synthesized and characterized as described previously [25]. The structures of synthesized 2-triazolylthioactamides are shown in Figure 1.

### 3.2. Inhibition of VIM-2 by 2-Triazolylthioactamides 

To evaluate 2-triazolylthioactamides, the VIM-2 was expressed and purified as previously described [21]. The steady-state kinetic assays were conducted on an Agilent UV8453 spectrometer using nitrocefin (60 µM) as a substrate and monitoring its hydrolytic product at 482 nm. 

**Figure 2:**
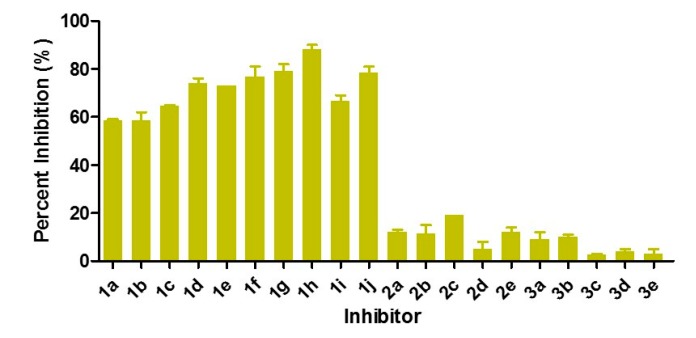
Percent inhibition of 2-triazolylthioactamides at a concentration of 100 μM against verona integron-encoded metallo-β-lactamase 2 (VIM-2) using nitrocefin as the substrate.

Percent inhibition of **1a**–**j**, **2a**–**e**, and **3a**–**e** (100 mM) against VIM-2 was determined and the results are displayed in Figure 2. It is clearly observed that **1a**–**j** showed more than 50% inhibition on VIM-2, while **1h** exhibited up to 90% inhibition. However, **2a**–**e** and **3a**–**e** with an aliphatic carboxyl had very weak inhibitory activity.

IC_50_ values of **1a**–**j** against VIM-2 were measured in 30 mM Tris, pH 7.6, in which the concentrations of inhibitors were varied between 0 and 120 µM. The IC_50_ data listed in Table 1 indicated that the 2-triazolylthioactamides **1a**–**j** inhibited VIM-2 with an IC_50_ value in the range 20.6–58.6 μM, and **1h** was the most potent inhibitor (IC_50_ = 20.6 μM). Moreover, clogP values of **1a**–**j** (Table 1), as predicted by the ALOGPS 2.1 program, are in the range 1.88–3.05, which are similar with many marked antibacterial agents [34,35].

Analysis of percent inhibition and IC_50_ data of the 2-triazolylthioactamides against VIM-2 revealed a structure−activity relationship (SAR), which is that the aromatic carboxyl substituted at triazole ring improves inhibitory activity of the thioacetamides against VIM-2. This SAR is similar to that in our previous reported on ImiS studies [25], hence it was significant to further probe the potential interaction of the aromatic carboxyl group to enzyme activity.

### 3.3. Antibacterial Activity Assays

The ability of 2-triazolylthioactamides to restore the antimicrobial activity of cefazolin against the bacteria producing VIM-2 was investigated by determining the MICs of β-lactams in the presence and absence of inhibitors (16 μg/mL). *E. coli* DH10B containing plasmid pBCSK-VIM-2 was used to study these inhibitors. A significant decrease in MIC of cefazolin (4–8-fold) was observed for **1a**–**j**, while **2a**–**e** and **3a**–**e** did not (Table 2A). The dose-dependent MIC assays for **1b**, **1c**, **1g**, and **1h** against *E. coli*-VIM-2 (Table 2B) indicated that the antibacterial effect of cefazolin increased gradually with the increasing inhibitor dose. In the presence of **1b**, **1c**, **1g**, or **1h** (128 μg/mL), the MICs of cefazolin were decreased 16-, 16-, 32-, and 32-fold, respectively. Although the strains treated with a high dose of 2-triazolylthioacetamides (128 μg/mL) were still resistant, they are promising in the future. Inhibitors alone did not show antibacterial activity against the *E. coli* and *E. coli* producing VIM-2 at 128 μg/mL, implying that the capacity of 2-triazolylthioacetamides to restore β-lactams activity lies in their inhibition of the MβLs harbored in bacteria.

### 3.4. Binding Affinity Evaluation

**Figure 3:**
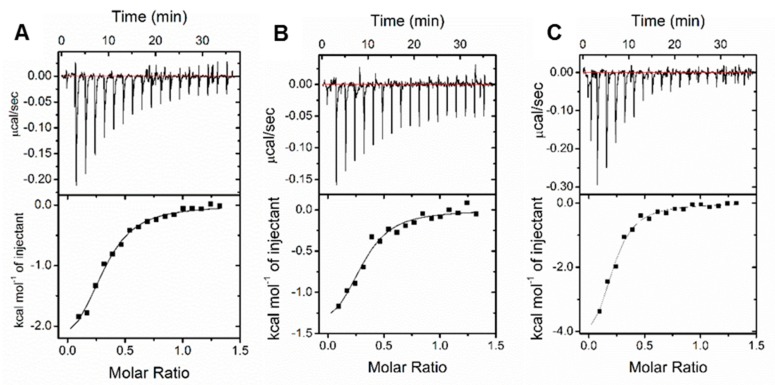
Isothermal titration calorimetry (ITC) thermograms of titration of **1b** (**A**), **1c** (**B**), or **1h** (**C**) solution (1 mM) into 100 μM VIM-2 in Tris-HCl, pH 7.6, 298 K (upper panels). The lower panels represent the integrated heat release of the titration after correcting dilution heat against the molar ration of **1b**, **1c**, or **1h**/VIM-2; the solid lines represent the best-fitted isotherm by the nonlinear fitting with the one-site model.

To investigate the binding affinity of 2-triazolylthioactamides to VIM-2, **1b**, **1c**, and **1h** as enzyme inhibitors were selected based on the inhibitory activity. ITC experiments were performed on MicroCal-ITC200 with a multiple injections mode at 298 K. Injection of the inhibitor into the protein solution was shown as the peak of the binding isotherm (upper panels, Figure 3), the bottom of Figure 3 displayed the integrated plots of heat released per injection as a function of protein-inhibitor molar ratio. After the best fitting by the single binding site model, the affinity constant (*K*_a_), corresponding dissociation constant (*K*_d_), and thermodynamic parameters (∆*H*, Δ*S*, and Δ*G*) were determined and listed in Table 3.

For the binding of **1b**, **1c**, and **1h** to VIM-2, the *K*_a_ and corresponding Δ*G* (∆**G = ∆**H − T∆**S) displayed in Table 3 revealed that the binding affinity of **1h** is higher than **1c**, and then **1b**, which is consistent with the IC_50_ values of these inhibitors against VIM-2 (20.6, 49.1, and 58.6 μM, respectively). These results implied that the initial binding **1b**, **1c**, or **1h** to VIM-2 was a combination of entropy and enthalpy contributions. Comparison of the structure of **1b** and **1c**, the NH group of **1b** improved the hydrophilicity and resulted in an increase of ∆*H* and a decrease of TΔ*S*. In addition, replacing the benzimidazole of **1b** or benzothiazole of **1c** with a chlorophenyl (**1h**) caused an obvious raising in ∆*H* and enhanced the inhibition of VIM-2, suggesting that the favorable enthalpic contributions which are assigned to van der Waals interaction, hydrogen bonding, or electrostatic interactions are the main driving force for improving the inhibitory activity of the 2-triazolylthioactamides on VIM-2 [36].

### 3.5. Crystallographic Analyses

**Figure 4:**
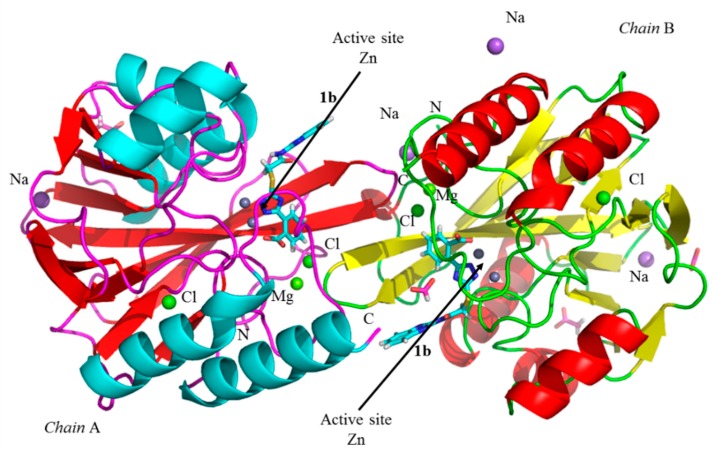
Two VIM-2 molecules in the asymmetric unit with a ribbon diagram. Helices were shown in cyan (chain A) or red (chain B) and β-strands are shown in red (chain A) or yellow (chain B). **1b** binding to the active site was shown in stick and colored by atom (H, white; C, cyan; N, blue; O, red; S, yellow). Zn^2+^, Cl^−^, Mg^2+^, and Na^+^ ions are shown as gray, green, yellow, and purple spheres, respectively.

**Figure 5:**
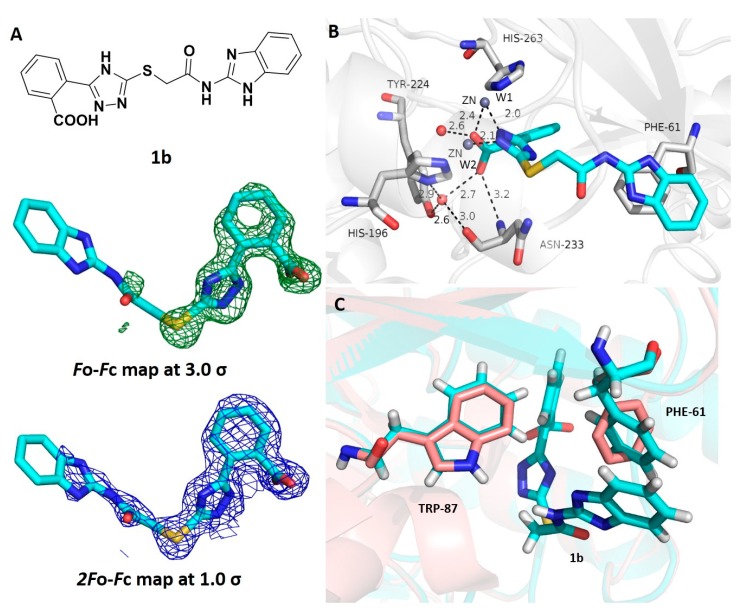
Crystallographic analyses of the binding mode of **1b** to VIM-2. (**A**) The ligand structure, *F*o-*F*c map was shown in green at 3.0 σ and final 2*F*o-*F*c density map in blue at 1.0σ, respectively; (**B**) crystal structure of compound **1b** bind to the activity site of VIM-2; (**C**) the structure alignment of native structure VIM-2 (pink, 1 ko3) and the complex structure of VIM-2 and **1b** (cyan, PDB: 6 KW1).

To explore how the 2-triazolylthioactamides bind to VIM-2, we attempted to obtain the X-ray crystal structure of VIM-2 in complexes with each **1a**–**j** compound. However, only the crystal structure of VIM-2 in complex with **1b** (**VIM-2:1b**) was obtained successfully through co-crystallization. Maybe the low solubility was the possible reason which resulted in other inhibitors without providing satisfactory complexes. **VIM-2:1b** was solved to 1.78 Å resolution (PDB ID: 6KW1), crystallized in space group C2 with two protein molecules in the asymmetric unit (ASU) in Figure 4, and refined to *R*_work_/*R*_free_ value of 17.65%/20.63%. The details for the collection and refinement statistics were listed in Table 4 and Table 5. The crystallographic analyses of the binding mode of **1b** to VIM-2 were shown in Figure 5. The OMIT *F*o-*F*c map and final 2*F*o-*F*c density map of ligand were shown at 3.0 and 1.0 sigma, respectively.

As is typical for B1 MβLs, there are two Zn(II) ions in the active sites of the enzyme. Zn1 was coordinated by His116, His118, while Zn2 was coordinated by Asp120, Cys221, and His263. In Figure 4, two Mg^2+^ were located between the two symmetry VIM-2 molecules, which has been observed in the published crystal structure of VIM-2 and considered to be a crystallographic artefact [8,37]. Moreover, two Cl^−^ and four Na^+^ ions have been observed. The protein active site structure, including Zn–ligand and the distances between the two Zn ions (3.9 Å) agreed with the publications [11,21,22,27].

The active sites structure of VIM-2 in complex with **1b** (Figure 5B) showed that the triazole ring of **1b** replaced the hydroxide and interacted with both Zn(II) ions (2.1 and 2.2 Å), which identified the previous SAR that the azolylthioactamides containing the triazole group was better for inhibition of NDM-1 (belong to the same subclass with VIM-2) than thiazole or oxazole substituted compounds [24]; the carboxyl moiety of **1b** interacted with Zn2 (2.4 Å), and formed H-bond with the backbone N atom of Asn233 (3.2 Å) on L10 loop; the O atoms of carboxyl interacted with the W1 (2.6 Å) and the structural water molecule W2 (2.7 Å) through hydrogen-binding, respectively, which was similar to the interactions for debris fastening with the active site of VIM-2 [11]. Importantly, we found that the phenyl at R_1_ position formed π-π stacking with the imidazole ring of His263, the favorable hydrophobic interaction reasonably explained the SAR that the aromatic carboxyl substituted 2-triazolylthioactamides (**1a**–**j**) exhibited a better inhibitory activity against VIM-2 than the aliphatic carboxyl substituted compounds (**2a**–**e** and **3a**–**e**). Moreover, Tony et al. reported that the binding of compound **1** (**1c**) caused a flip of the backbone and side-chain of Trp87 and believed that the conformational changes of Trp87 were important to further optimize triazolylthioacetamide inhibitors [27]. In our crystal structure, residues Trp87 kept a native pose, yet the binding of **1b** to the active sites of VIM-2 resulted in a conformational change of the residue Phe61 on the L3 loop and formed a potential clash with Phe61 utilizing the benzimidazole group (Figure 5C).

## 4. Conclusions

The 2-triazolylthioacetamides **1a**–**j, 2a**–**e**, and **3a**–**e** were synthesized, and kinetic assays showed that compounds **1a**–**j** were potent inhibitors of VIM-2 with an IC_50_ value in the range 20.6–58.6 μM, and **1h** was found to be the best inhibitor (IC_50_ = 20.6 μM). SAR studies revealed that the substituted aromatic carboxyl at triazole ring improved inhibition of the molecules on VIM-2. MIC tests indicated that **1a**–**j** partly restored the antimicrobial effect of cefazolin against *E. coli* expressing VIM-2, although the strains treated with a high dose of 2-triazolylthioacetamides (128 μg/mL) were still resistant. Thermodynamic characterization suggested that the binding of **1b**, **1c**, or **1h** to VIM-2 was a combination of entropy and enthalpy contributions, and replacing the bulky hydrophobic group with a small hydrophobic group at R_2_ improved inhibition of 2-triazolylthioactamide on VIM-2. The crystallographic analysis revealed that **1b** replaced the hydroxide which bridges the two Zn(II) ions and bound to the Zn(II) ions via N atoms on the triazole ring; the carboxyl moiety of **1b** interacted with Zn2 and formed an H-bond with the backbone N atom of Asn233 and the water molecule W2. Although the crystal structure in the complex with 2-triazolylthioacetamide has been reported [27], we proved the importance of phenyl at R_1_ to the inhibitory activity of 2-triazolylthioactamide against VIM-2, which formed a hydrophobic interaction with active residue His263 and confirmed the above SAR; further, a new hydrophobic interaction has been found where the benzimidazole formed a potential disturbed with Phe61 and resulted in a conformational change of the residue Phe61 on the L3 loop. This work revealed that the aromatic carboxyl at R_1_ was essential for the activity of inhibitors on VIM-2, further, the *m*-chlorophenyl at R_2_ promoted activity of the compounds on VIM-2. The information gained in this work is valuable for further optimizing 2-triazolylthioacetamides as VIM-2, even as broad-spectrum MβLs inhibitors.

## Figures and Tables

**Table 1:** The inhibition of VIM-2 (IC_50_), and clogP values of aromatic carboxyl substituted 2-triazolylthioactamides **1a**–**j**.

Compds	IC_50_ (μM)	clogP	Compds	IC_50_ (μM)	clogP
**1a**	40.8 ± 0.3	2.28	**1f**	45.8 ± 0.2	1.88
**1b**	58.6 ± 0.1	2.18	**1g**	31.4± 0.4	3.03
**1c**	49.1 ± 0.1	2.74	**1h**	20.6 ± 0.1	3.05
**1d**	52.2 ± 0.5	3.05	**1i**	45.2 ± 0.1	2.14
**1e**	38.2 ± 0.3	2.23	**1j**	57.6 ± 0.4	2.19

The substrate was nitrocefin and inhibitor concentrations were varied between 0 and 120 μM; data were mean ± standard.

**Table 2:** Minimum inhibitory concentration (MICsμg/mL) of cefazolin against *E. coli* DH10B-VIM-2 in the absence and presence of 2-triazolylthioactamides at 16 μg/mL (**A**), and in the presence of **1b**, **1c**, **1g**, and **1h** in a concentration range of 0–128 μg/mL (**B**).

**(A)**							
**Compds**		**MIC (μg/mL)**		**Compds**		**MIC (μg/mL)**	
Control		128		**1f**		32	
**1a**		32		**1g**		16	
**1b**		32		**1h**		16	
**1c**		32		**1i**		32	
**1d**		32		**1j**		16	
**1e**		16					
**(B)**							
**Compds**\conc	0		16	32	64		128
**1b**	128		32	32	16		8
**1c**	128		32	32	16		8
**1g**	128		16	16	8		4
**1h**	128		16	8	8		4

The MICs of cefazolin against *E. coli* cells without VIM-2 was 1 μg/mL.

**Table 3:** Binding abilities and thermodynamic parameters of **1b**, **1c**, or **1h** to VIM-2, as estimated by ITC at 298 K in Tris-HCl, pH 7.6.

Compounds	*K*_a_ (M^−1^)	*K*_d_ (μM)	Δ*H* (kcal/mol)	TΔ*S* (kcal/mol)	Δ*G* (kcal/mol)
**1b**	1.16 × 10^5^	8.62	−2.66	4.26	−6.92
**1c**	1.26 × 10^5^	7.94	−1.61	5.34	−6.95
**1h**	1.56 × 10^5^	6.41	−5.10	1.98	−7.08

**Table 4:** Data collection.

Structure (PDB ID)	6A61
*Data Collection*	SSRF BL18u
Space group	*C* 1 2 1
a, b, c (Å)	103.759, 79.421, 67,981
α, β, γ (°)	90, 130.938, 90
*^a^* Mol/ASU	2
Resolution range (Å)	39.19–1.78 (1.839–1.78)
Number of unique reflections	39948
Completeness (%)	99.13
I/σ(I)	6.3 (3.8)
Wilson B Factor (Å^2^)	16.10

*^a^* Mol/ASU: Molecules per asymmetric unit.

**Table 5:** Refinement statistics.

Overall B factor (Å^2^)	24.15
Protein B factor (Å^2^)	22.80
Ligand B factor (Å^2^)	50.62
Water B factor (Å^2^)	31.79
*^b^* RMSD from Ideal Bond Length (Å)	0.006
*^b^* RMSD from Ideal Angles (°)	0.83
Final R_work_ (%)	17.65%
Final R_free_ (%)	20.63%

*^b^* RMSD: Root mean square deviation.
